# Bio-Molecular Applications of Recent Developments in Optical Tweezers

**DOI:** 10.3390/biom9010023

**Published:** 2019-01-11

**Authors:** Dhawal Choudhary, Alessandro Mossa, Milind Jadhav, Ciro Cecconi

**Affiliations:** 1 Department of Physics, Informatics and Mathematics, University of Modena and Reggio Emilia, 41125 Modena, Italy; dhawal.choudhary@unimore.it (D.C.); msjadhav020@gmail.com (M.J.); 2 Center S3, CNR Institute Nanoscience, Via Campi 213/A, 41125 Modena, Italy; 3 Istituto Statale di Istruzione Superiore “Leonardo da Vinci”, Via del Terzolle 91, 50127 Firenze, Italy; 4 Istituto Nazionale di Fisica Nucleare, Sezione di Firenze, Via Giovanni Sansone 1, 50019 Sesto Fiorentino, Italy

**Keywords:** plasmonic optical tweezers, femtosecond optical tweezers, photonic crystal optical tweezers, fluorescence, single molecule and cell studies

## Abstract

In the past three decades, the ability to optically manipulate biomolecules has spurred a new era of medical and biophysical research. Optical tweezers (OT) have enabled experimenters to trap, sort, and probe cells, as well as discern the structural dynamics of proteins and nucleic acids at single molecule level. The steady improvement in OT’s resolving power has progressively pushed the envelope of their applications; there are, however, some inherent limitations that are prompting researchers to look for alternatives to the conventional techniques. To begin with, OT are restricted by their one-dimensional approach, which makes it difficult to conjure an exhaustive three-dimensional picture of biological systems. The high-intensity trapping laser can damage biological samples, a fact that restricts the feasibility of in vivo applications. Finally, direct manipulation of biological matter at nanometer scale remains a significant challenge for conventional OT. A significant amount of literature has been dedicated in the last 10 years to address the aforementioned shortcomings. Innovations in laser technology and advances in various other spheres of applied physics have been capitalized upon to evolve the next generation OT systems. In this review, we elucidate a few of these developments, with particular focus on their biological applications. The manipulation of nanoscopic objects has been achieved by means of plasmonic optical tweezers (POT), which utilize localized surface plasmons to generate optical traps with enhanced trapping potential, and photonic crystal optical tweezers (PhC OT), which attain the same goal by employing different photonic crystal geometries. Femtosecond optical tweezers (fs OT), constructed by replacing the continuous wave (cw) laser source with a femtosecond laser, promise to greatly reduce the damage to living samples. Finally, one way to transcend the one-dimensional nature of the data gained by OT is to couple them to the other large family of single molecule tools, i.e., fluorescence-based imaging techniques. We discuss the distinct advantages of the aforementioned techniques as well as the alternative experimental perspective they provide in comparison to conventional OT.

## 1. Introduction

Optical tweezers (OT) technology has emerged as a prime tool for biological research over the last three decades, ever since the seminal works by Ashkin and co-authors [1,2,3]. The ability of light to exert force on matter is exploited by OT to precisely and noninvasively manipulate target molecules. Since its origins, this technique has experienced a dramatic evolution that has increasingly broadened its range of applications in biophysics. Thanks to the development of accurate manipulation techniques [4,5,6,7], advanced strategies to reduce mechanical and thermal noise [8], and increasingly sophisticated spatial and temporal detection methods [9,10], it is nowadays possible to use optical tweezers to measure piconewton forces and up to angstrom-level displacements [11]. Parallel and in close connection to the instrumental developments, recent breakthroughs in statistical mechanics have coalesced into the field called stochastic thermodynamics [12,13] which is now accepted as the physical framework to interpret experimental data. These technical and theoretical advances have allowed biophysicists to employ optical tweezers in the investigation of an increasing number of biological processes, including the mechanochemistry of molecular motors [14,15,16,17]; the torsional and bending rigidity of biopolymers [18]; the noncovalent binding interactions between ligand–receptor pairs [19]; and the mechanical unfolding, refolding, and misfolding [20,21,22] of proteins [23,24,25,26], DNA [27,28,29,30,31], and RNA [32,33] molecules.

The further expansion of the already broad spectrum of OT’s applications is however restricted due to certain experimental limitations. It is still very difficult, for example, with conventional optical tweezers to directly trap nanometer-sized targets. Conventional OT are also ill-equipped for providing detailed conformational and chemical change analysis about complex biomolecular systems. Moreover, cell manipulation via lasers is often accompanied by photo damage, which narrows the scope of such experiments. A considerable effort has been devoted to overcome the restrictions on conventional OT [34,35], developing new instrumental setups by inventively combining optical traps with spectroscopy [36], microfluidics [37], acoustics [38], plasmonics [39,40], and photonics [41], to name a few. The next generation versions of optical tweezers boast an expansive literature and have paved the way for new perspectives in optical manipulation and single molecule studies. This review narrows its focus down to four variations of conventional optical tweezers specifically designed to address the aforementioned limitations namely plasmonic optical tweezers (POT), photonic crystal optical tweezers (PhC OT), femtosecond optical tweezers (fs OT), and optical tweezers combined with various fluorescence techniques. In particular, it will provide a description of the main instrumental features, biological applications, and further scope of these techniques.

## 2. Plasmonic Optical Tweezers

A major limitation of conventional optical tweezers is their inability to manipulate directly minuscule biomolecules, such as DNA, RNA, and proteins. This limitation can be easily understood if we consider that, when the radius *r* of the particle is much smaller than the wavelength of the light (Rayleigh regime), the trapping potential (U) generated by a laser beam is given by [42]:

(1) $$U\approx -\int F_{trap}dx=-\int (\left|\alpha \right|/2)\nabla \langle E^{2}\rangle dx=-\left(\left|\alpha \right|/2\right)\langle E^{2}\rangle ,$$

where $F_{trap}$ is the trapping force experienced by the particle, $\langle E^{2}\rangle$ is the time averaged square of the electric field, and α is the polarizability of the target particle, which depends on its volume ($V=4\pi r^{3}/3$) according to the relation:

(2) $$\alpha =3V\frac{\epsilon_{p}-\epsilon_{m}}{\epsilon_{p}+2\epsilon_{m}},$$

where $\epsilon_{p}$ and $\epsilon_{m}$ are the dielectric constants of the particle and the medium, respectively. In order to achieve a stable trapping, *U* must be much higher than the thermal fluctuations of the particle in the medium, that is [2,43]:

(3) $$\left|U\right|\gg K_{B}T,$$

where *K_B_* is the Boltzmann constant and *T* the absolute temperature. For a nanometer-sized particle α becomes very small, and so does *U*. As a consequence, Equation (3) does not hold true and the particle escapes the optical trap.

The observation that a larger trapping force can be achieved in regions where the electric field is rapidly varying led to the development of near-field optical microscopy [44]. In the presence of laser illumination, the free electron density on the surface of a metal can undergo collective oscillations that result in standing waves called plasmons. Under these conditions, the charge concentration due to geometrical features like tips or sharp edges creates an evanescent field whose intensity decreases very fast with the distance from the conductor surface. The field enhancement due to localized surface plasmons has opened up new possibilities for optical trapping at the nanometer scale [44]. Based on this principle, a novel technique for optical manipulation has recently been developed, which has been called plasmonic optical tweezers (POT) [39,42,43]. Improved optical trapping is achieved in POT using a subwavelength concentration of propagating laser light into plasmonic hotspots composed of evanescent field with high localized intensity. Expertly fabricated plasmonic nanostructures are used as a conduit to create such plasmonic hotspots, thereby strongly enhancing the electric field of the incident laser light. The increased electric filed compensates for the low polarizability of nanometer-sized particles, resulting in a strong trapping potential. Thus Equation (3) is re-established in POT, allowing for the trapping and manipulation of miniscule particles.

The realization of three-dimensional plasmonic optical traps was first reported by Grigorenko et al. [39]. The authors used a focused 1604 nm diode laser beam to generate plasmonic optical traps in proximity of closely spaced pairs of gold nanodots. The near-fields of the nanodot pairs produced subwavelength trapping volumes where nanometer-sized polystyrene beads could be stably captured. The large stiffness of the potential well of the plasmonic trap significantly quenches the Brownian motion of the captured object, dramatically improving particle positioning as compared to that normally achieved with conventional diffraction-limited optical traps. Escape velocity measurements showed large trapping forces, even with low laser powers, opening up new exciting possibilities for the nanomanipulation of biological samples with plasmonic trapping.

After the pioneering work of Grigorenko et al. [39], new experimental approaches were designed to improve POT. Thermal energy released due to ohmic losses during plasmon excitation can trigger overheating of water, generation of steam bubbles, and thermal convection. To counter these overheating problems, Wang et al. [45] exploited the high thermal conductive properties of copper and silicon. Through simple nanofabrication techniques, the authors generated nanopillars on a gold film deposited on a copper layer in contact with a silicon substrate. During plasmonic trapping, copper and silicon act as a heat-sink conducting the heat from the nanopillar to the substrate, thus minimizing (the generated heat in) water warming. Using these nanostructures, the authors were able to stably trap 110 nm beads and rotate them upon manual rotation of the incident linear polarization (*λ* = 974 nm). On the other hand, when circularly polarized light was used for trapping, the captured beads could be passively rotated clockwise or counterclockwise by changing the handedness of the incident light, demonstrating precise control gained at nanoscale through plasmonic optical trapping.

**Figure 1:**
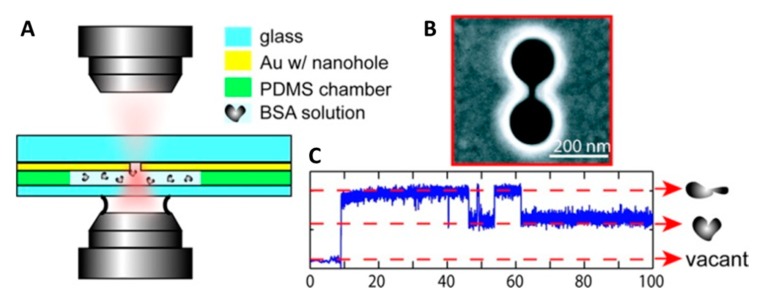
Double-nanohole optical tweezers. (**A**) An 820 nm laser is focused with a 100× oil immersion microscope objective near a double-nanohole milled on a commercially available 100 nm thick Au film. The plasmonic trap is generated inside a polydimethylsiloxane (PDMS) microwell filled with a 1% bovine serum albumin (BSA) solution (*w/w*) in phosphate-buffered saline (PBS). (**B**) Scanning electron microscope image of the double-nanohole fabricated using a focused ion beam (FIB). The double-nanohole is characterized by two sharp tips (cusps) separated by 15 nm. (**C**) Time trace of the optical power transmitted through the double-nanohole. Upon trapping of a BSA molecule, the optical power sharply increases and then fluctuates between two distinct levels as the molecules hops between its folded and unfolded states. Reprinted with permission from [51]. Copyright 2012 American Chemical Society.

Direct trapping of smaller and smaller particles was then achieved by employing nanostructures with varying morphologies, such as nanoapertures in gold film [46], double-nanoholes (DNH) [47] and nanodipole antennas [48]. Righini et al. [49] further extended the work done in [39] by developing plasmonic optical traps between gold nanoantennas employing laser light with three times less intensity. Significantly, not only were they able to trap polystyrene beads of variable size ranging from 1 µm to 200 nm but also successfully demonstrated the trapping of *Escherichia coli* bacteria for the first time. The elongated cells of *E. coli* bacteria were immobilized in a stable horizontal geometry in the optical trap by focusing a Ti:sapphire laser with a wavelength of 800 nm on an array of gold nanoantennas spaced by a distance of 30 nm. The authors also studied the growth of the optically trapped cells and these cells were shown to grow and divide on a time scale similar to the cells that were not trapped, signifying lack of significant photodamage to cells due to optical trapping. Similarly, Huang et al. [50] were able to trap yeast cells (*Saccharomyces cerevisiae*) by combing plasmonic optical traps with microfluidics. These results open considerable avenues for application of POT in biological matter manipulation. In 2011, POT was used to trap single protein molecules [51]. Double-nanoholes milled on thin gold films were employed by Pang et al. [47] to generate plasmonic traps capable of stably trapping single bovine serum albumin (BSA) molecules, Figure 1. The trapping of a single protein could easily be detected by the instrument and the fate of the captured molecule could be followed with high-resolution over an extended period of time. Under the action of the optical forces, a trapped BSA molecule readily denatures and then hops between its folded and unfolded states, giving rise to two distinct levels of optical power in the recorded traces (Figure 1C). The ease with which the DNH optical tweezers reveals the trapping of a single BSA molecule has prompted the authors to hypothesize a possible development of the instrument in a biosensor for single protein detection.

A few years later, the same experimental group used DNH optical tweezers to trap single 10 bp DNA hairpins and study their interaction with the tumor suppressor p53 protein [52]. The authors showed that after trapping, the optical forces of a plasmonic trap unzip a 10 bp DNA hairpin in a time scale of ≈0.1 s. If the same experiment is performed in the presence of p53, the unzipping time becomes longer as the binding of p53 to the DNA stabilizes the hairpin, increasing its unfolding activation energy by ≈2 × 10^−20^ J. No unzipping delay is instead observed in the presence of the p53 mutant (Cys135Ser), revealing the inability of this protein to suppress the denaturation of a DNA hairpin and providing a possible molecular-level explanation for the ineffectiveness of p53 in suppressing tumor growth. Although the work presented in [52] does not provide definitive results on the mechanism of interaction between DNA and p53, it certainly shows the capability of DNH optical tweezers to study directly the interaction between proteins and short DNA molecules, without the need to use molecular handles and/or fluorescence labels as done typically in experiments performed with conventional optical tweezers.

## 3. Photonic Crystal Optical Tweezers

The same idea of using evanescent near-fields that allow POT to trap objects well below the diffraction limit has promoted the development of slot waveguides [53] and photonic crystal traps [35,54]. Light propagating via total internal reflection into a waveguide generates an evanescent field on the outside whose intensity decays exponentially with the distance from the waveguide surface. This near-field can be used to transport a dielectric particle along the waveguide, while a fixed-position trap can be realized by placing a photonic crystal resonator along the track, demonstrating a new class of optical trap, termed PhC OT [55]. Photonic crystals (PhC) [56,57,58] can be described as structures with a periodic pattern in the dielectric properties. In close analogy with ordinary crystals, PhC are characterized by photonic band gaps, which allow or forbid light in a certain frequency range to pass through.

**Figure 2:**
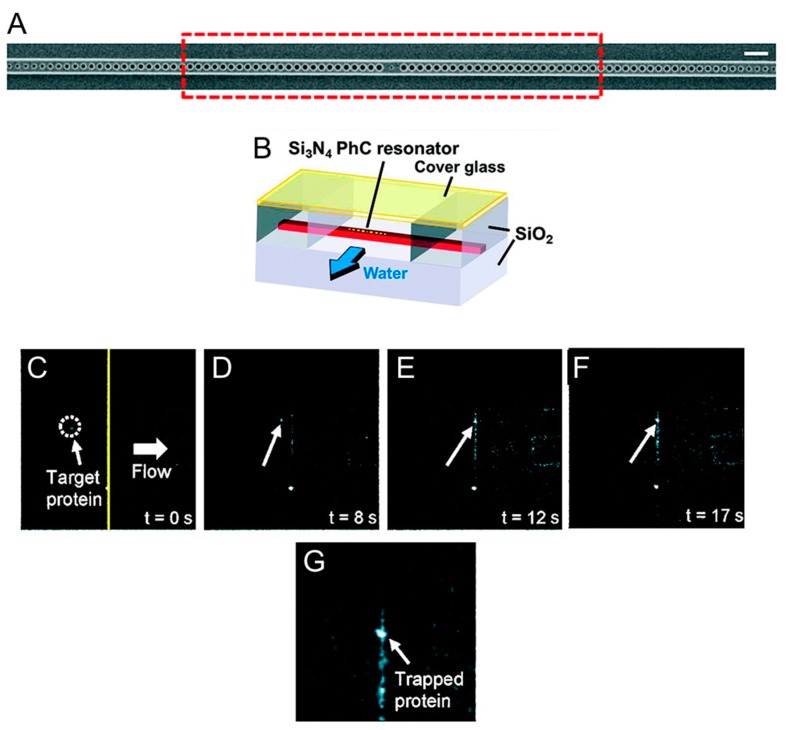
Trapping of individual proteins with a photonic crystal resonator. (**A**) Scanning electron microscope image of the silicon nitrite photonic crystal resonator fabricated by Chen et al. [41] to trap nanometer-sized objects. The resonator consists of a central hole flanked by 53 holes on both sides and is operated at 1064 nm. Scale bar: 1 µm. (**B**) Schematic of the flow chamber used to flow protein molecules near the resonator. (**C**–**F**) Fluorescence microscope images of Cy5-labeled Wilson disease proteins inside the flow chamber. As the flow is activated (*t* = 0 s), the proteins that are conveyed near the optically excited resonator remain stably trapped. A high-resolution picture of a group of trapped proteins is shown in (**G**). Adapted with permission from [41]. Copyright 2012 American Chemical Society.

The biological applications of photonic crystals are not yet plentiful but have already highlighted the advantages that this technique presents compared to traditional optical tweezers, especially with regard to the ability to trap biological material without damaging it. Chen et al. [41] have designed special photonic crystal resonators capable of trapping nanometer-sized objects with minimal temperature increase (Figure 2). They fabricated a small hole in the center of the cavity and manufacture the device using silicon nitride in order to operate the photonic crystal resonator at 1064 nm [59]. At this wavelength the optical absorption of water is reduced by two orders of magnitude compared to that measured at 1550 nm, the resonant wavelength of silicon resonators, and thus local warming at the resonator cavity is reduced [41,60]. Using these devices, Chen et al. [41] managed to trap nanometer-sized objects with a local temperature rise of less than 0.3 K, thus minimizing the possibility of damaging biological matter. Figure 2 shows the optical trapping of a truncated version of the Wilson disease protein (molecular weight ≈46 kDa). When proteins in solution pass near the optically excited resonator they remain stably trapped. This process is reversible as the proteins return to being free in solution as the 1064 nm laser is switched off. Using the silicon nitride PhC resonators fabricated by Chen et al. [41], it is thus possible to confine biomolecules reversibly in subwavelength trapping volumes without subjecting them to temperature increases that would compromise their functionality. This opens up new perspectives for further studies of the interactions between proteins and their targets (other proteins, DNA fragments, or nanoparticles), and for facilitating assembly of nanometer-sized biomaterial. More recently, photonic crystals have also been employed to optically trap eukaryotic and prokaryotic cells [61]. By focusing a continuous wave (cw) 1060 nm laser on the surface of two-dimensional photonic crystals, Jing et al. were able to create low intensity optical traps where they could stably capture living cells. By analyzing the morphology of the cells and the cellular adsorption of propidium iodide over time [62], the authors showed that trapped NIH/3T3 mammalian fibroblasts maintain their viability for more than 30 min, while *E. coli* cells can complete their 20 min life cycles. Being able to extend the viability of optically trapped cells, the PhC OT described in [61] have great potential for various biomedical applications, including the characterization of the mechanical properties of cells [63] or the study of the relation between mass and cell growth.

## 4. Femtosecond Optical Tweezers

A recent evolution in instrumental design for optical trapping technique has been the replacement of continuous-wave laser sources with ultrashort femtosecond lasers. Optical tweezers using cw laser sources are characterized by their constant trapping force. A femtosecond (fs) laser source on the other hand allows for different dynamics in optical trapping as the target is put through spontaneous impulsive cycles of drag and release [64]. These drag and release cycles are due to the nature of fs lasers that produce a constant stream of laser pulses with high peak power followed by brief intervals where no force is experienced by the target. During the laser pulses, extremely high photon pressure is induced by fs laser sources, which is approximately 10^5^ times higher than that of a cw laser with same power [65]. Under these conditions, in order to achieve stable trapping, the diffusion experienced by the target during a no pulse interval due to gravitational and Brownian forces must be countered by the optical force acting on the particle during a high peak pulse [66]. As shown by Xing et al. [66], the diffusion velocity $v_{1}$ of a spherical target particle of radius *r* and volume *V* during the no pulse interval *t* is dominated by the competition between gravity and buoyancy, so it is given by [66]:

(4) $$v_{1}=\left(\rho_{p}-\rho_{m}\right)gV/\left(6\pi \mu r\right),$$

where g is the acceleration due to gravity, μ is the liquid viscosity, and $\rho_{p}$ and $\rho_{m}$ are the densities of the particles and the medium, respectively. It follows that the displacement of the particle during the no pulse interval is:

(5) $$s_{1}=v_{1}t.$$

On the other hand, during the pulse interval$\;t_{0}$, the optical trapping force F will drag the particle back towards the trap with a velocity $v_{2}$ given by:

(6) $$v_{2}=\left[F-\left(\rho_{p}-\rho_{m}\right)gV\right]/\left(6\pi \mu r\right),$$

and the displacement of the particle will be:

(7) $$s_{2}=v_{2}t_{0}.$$

For stable trapping with a femtosecond laser the restoring displacement $s_{2}$ must be larger than the falling displacement $s_{1}$, and thus the following equation must hold true:

(8) $$\;F\geq \left(\rho_{p}-\rho_{m}\right)gV\left[\left(t+t_{0}\right)/t_{0}\right].$$

This stability condition can be achieved both in the Rayleigh regime [67] (trapped particle much smaller than the trapping wavelength) and the Lorenz–Mie regime [68] (trapping wavelength and trapped particle size are of the same order of magnitude). Malmqvist and Hertz were the first to report simultaneous optical trapping of and second-harmonic generation in 50–100 nm particles using a 1.06 μm fs laser [67], while Agate et al. [68] were the first to demonstrate femtosecond optical tweezers (fs OT) in the Lorenz–Mie regime. The authors used a titanium-sapphire near infrared (NIR) fs laser to stably trap ≈1 um beads and measured the lateral trapping force (Q-value) acting on the captured bead to demonstrate that the tweezing action of an fs laser can be as effective as that of a cw laser with the same average power. Moreover, and importantly, Agate et al. [68] showed that an fs laser can be used for simultaneous trapping and multiphoton absorption of a target particle. The impulsive pulses of an fs laser rapidly release energy resulting in extremely high peak powers. The light intensity of such a pulse is in the range of 10^5^ W/cm^2^ and the peak power can be as high as a few megawatts. The interaction of such high intensity high peak power laser with material induces deviation from classical behavior, enabling the exploration of nonlinear processes such as multiphoton absorption [64]. Using an fs laser operating at 800 nm, Agate et al. [68] achieved two-photon excitation of optically trapped dye-doped polymer particles absorbing at 400 nm and emitting at ≈450 nm, and showed how the fluorescent light could be turned on and off reversibly by simply switching the laser source between the fs and the cw regimes, with no effect on tweezing efficiency. The possibility of switching on and off the light emitted by a trapped particle is a unique feature of femtosecond OT that might have interesting developments for the manipulation and visualization of biological samples. A better theoretical understanding of the roles played in optical trapping by the various facets of fs lasers, such as pulse width, pulse repetition rate, transient forces, and multiphoton absorptions will help us devise better fs OT systems [69,70].

**Figure 3:**
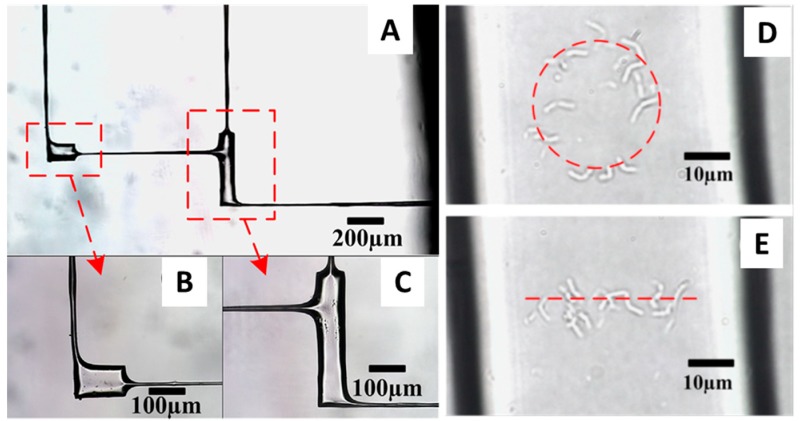
(**A**) Microfluidic device made of two micropools and four microchannels. (**B**,**C**) Magnified views of the micropools. (**D**,**E**) Circular and linear distributions of *B. subtilis* manipulated inside the micropools with a fs laser trap. Adapted with permission from [73]. Copyright 2014 Elsevier.

The biological applications of fs lasers have been limited to date and confined mostly, although not exclusively, to the manipulation of cells. Yet, the importance of fs lasers in biology is bound to increase as some intrinsic features of these lasers make them particularly well suited for the manipulation of soft matter. Femtosecond lasers have high peak power, but their short pulse duration prevents the mode-locked laser oscillators to generate a population inversion at high energies, limiting the pulse energy to a few nanojoules. As a consequence, fs lasers operate in a weak energy regime, making them a viable tool for the manipulation of cells. Zhou et al. [71], used a 800 nm femtosecond Ti:sapphire laser to stably trap human red blood cells (hRBCs), and showed the ability to make them rotate inside the trap by modulating the laser light intensity. Stable optical trapping of hRBCs with fs lasers was also achieved by Mao et al. [72] who also measured the escape velocity of these cells from the optical traps to show a tweezing efficiency comparable to that of cw lasers. No cell damage was reported in these studies as well as in the study presented by Li et al. [73], where the authors used fs lasers to trap living prokaryotic cells. Li et al. [73] first performed a water-assisted fs laser ablation of the internal surface of a microfluidic device to make it less rough and thus improve its optical properties (Figure 3). Then they used the same fs laser to trap single *Bacillus subtilis* cells and move them from one micropool to another through microchannels of 10 μm diameter. Finally, they manipulated individual *B. subtilis* inside the micropools to distribute them according to specific patterns. The results of these studies reveal the great potential that the combination microfluidic devices and fs laser OT might have for living cell sorting and manipulation.

**Figure 4:**
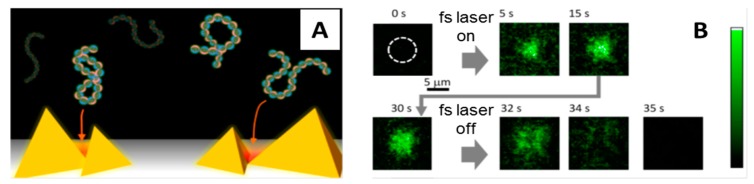
(**A**) Illustration showing gold nanopyramidal dimers and DNA molecules being trapped in plasmonic traps (red areas). (**B**) Fluorescence micrographs showing assembly of λ-DNA molecules stained with YOYO-1 in the area of the plasmonic substrate irradiated with the femtosecond (fs) laser (dashed white circle). As the fs laser is turned on, the irradiated area becomes brighter and brighter over time as an increasing number of molecules are trapped. When the laser is turned off the molecules go back in solution. Adapted with permission from [75]. Copyright 2013 American Chemical Society.

Femtosecond lasers have also been used in POT to trap biomolecules. Roxworthy and Toussiant [74] were the first to report on efficient trapping of nanosized objects using POT incorporated with fs lasers. They used a 100 fs Ti:sapphire (800 nm) laser and gold bowtie nanoantenna arrays to create plasmonic traps that stably captured silver nanoparticles (80 nm), reporting a two- and five-fold increase in trap stiffness compared to cw laser plasmonic traps and conventional OT traps, respectively. Similarly, Shoji et al. [75] used an fs NIR laser to create plasmonic traps on gold nanopyramidal arrays for capturing λ-DNA molecules stained with YOYO-1 (Figure 4). When they focused the laser on the plasmonic substrate, they observed accumulation of the DNA molecules in the irradiated area that under a fluorescent microscope becomes brighter and brighter over time as more and more molecules are trapped. Upon switching off the laser light the molecules rapidly go back in solution. The authors trapped λ-DNA molecules on the same plasmonic substrate also using a cw laser, but in this case the trapping process was not reversible as the molecules remained stuck on the gold surface when the cw laser irradiation was turned off. Why reversible trapping and release of DNA is possible only with fs lasers is not clear, but these results surely reveal important differences between fs and cw plasmonic traps that should be understood better to exploit them efficiently for the manipulation of other biomolecules.

## 5. Optical Tweezers Combined with Fluorescence

An obvious limitation of optical tweezers is that they provide one-dimensional data: we can measure the molecular extension only along the direction of the applied force. The experimenter is therefore compelled to adopt as a reaction coordinate the end-to-end extension, a choice which could miss important details of the system under investigation [76,77]. There are ways to circumvent this obstacle by adopting special experimental configurations that allow simultaneous manipulation along different directions: for instance, the Q-trap consisting of four independently controlled optical traps [78], or the DNA Y structure whose trunk is anchored to a microscope coverslip while the two arms are manipulated by a dual trap [79]. The peculiar geometry severely limits the kind of systems that can be studied by these means. In a more generally applicable approach, one can resort to repeated experiments, where handles are attached to different sites of the same molecule [80,81], but this is hardly a satisfying solution. On their own, optical tweezers remain blind to chemical and structural changes that do not produce a measurable variation of the molecular extension in the one direction susceptible to measurement.

It is therefore quite natural to try and complement the strength of optical tweezers by marrying them with the other great family of single-molecule experimental methods: those based on fluorescence properties [82]. From the simple localization of a single fluorophore to the high-resolution detection of Förster resonance energy transfer (FRET) [83], fluorescence-based methods add visualization capabilities to the mechanical manipulation skills of OT, the result being a very powerful single-molecule technique whose potential has just begun to be explored. A few recent reviews [84,85,86] make an excellent job of detailing the earlier successes and the state of the art of such hybrid experimental setups. Here, we limit ourselves to a very concise sketch of the most promising instrumental developments and biological applications, with special attention to the latest results.

Any attempt to combine fluorescence and optical manipulation runs into two main technical challenges: (1) the weak fluorescence signal must be efficiently isolated from other light sources (bright-field illumination, background fluorescence, the same trapping laser); and (2) the high-intensity laser used for the trap hastens the phenomenon of photobleaching [87]. Regarding the first issue, there are, broadly speaking, two traditional approaches to fluorescence spectroscopy, namely wide-field and confocal, and they offer a trade-off between ease of visualization and absence of background luminosity. As for the photobleaching problem, three conceptually different solutions have been successfully essayed: clever fine-tuning of the optical properties of the setup, and separation between fluorescence and trapping either in space, or in time. Finally, it is possible to add fluorescence detection to several OT setups: single- or dual (or multi-) trap, with the option of using the coverslip as an additional tethering point.

In the following we will show a selection of recent literature that covers many of the variations in the experimental setup and the biological system under study. Before that, however, it may be useful to give a quick rundown on the terminology of fluorescence techniques. We refer to epi-illumination when the light used for the excitation of the fluorophore goes through the same objective lens that is used for focusing the fluorescence to the observer. As all the fluorescence produced within the sample is detected, unwanted background fluorescence limits the concentration of fluorophores at which it is still possible to resolve a single emitter. The desire to reduce the background luminosity leads to the introduction of the total internal reflection fluorescence (TIRF): fluorophores are excited by an evanescent field that decays exponentially with the distance from the reflecting surface, thus limiting the field of view to a narrow (no more than 200 nm) region close to the coverslip. Both epi-illumination and TIRF are widefield techniques, offering direct access to a widefield of view.

A drastic solution to the background problem is to let the fluorescent light reach the detector through a pinhole that blocks most of the out-of-focus signal. This is the principle of confocal microscopy. One common use of the confocal setup is to detect FRET [88,89]. The efficiency of the energy transfer between two chromophores is an extremely sensitive measure of the distance between them, providing subnanometer details about spatial rearrangements undergone by the molecule object of investigation. As the field of view in confocal microscopy is limited to a very small region (ideally just a spot), a complete image of the sample can be obtained only by scanning, i.e., moving the confocal point within a grid of predefined positions. This has the advantage that a three-dimensional image can be reconstructed, albeit with reduced temporal resolution, and the disadvantage of accelerated photobleaching per detected photon [90], due to the fact that also out-of-focus portions of the sample are submitted to long exposure time while only a fraction of the fluorescence actually reaches the detector. This can be avoided by switching from single-photon to two-photon excitation (TPE) fluorescence: two lower energy photons are simultaneously (i.e., within one attosecond) absorbed by the fluorophore, which then releases a shorter wavelength photon. The cross-section for two-photon absorption is small, so a powerful excitation laser is needed, but the illumination is efficiently concentrated (both in time and space), so there is no out-of-focus photobleaching and the lifetime of fluorophores results are actually longer.

**Figure 5:**
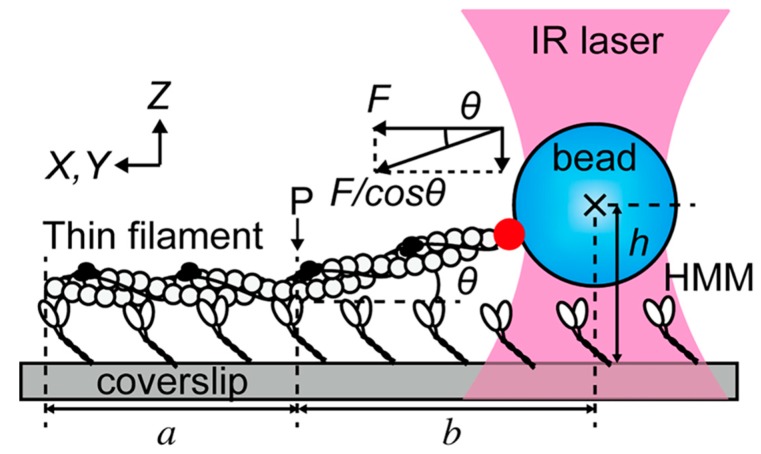
Experimental setup used by Ishii et al. [91]. The filament made of actin, tropomyosin, and troponin is attached to the coverslip for a length *a* up to the point *P* by its interaction with heavy meromyosin molecules (HMM). One end is connected to a polystyrene bead trapped by an infrared (IR) laser. The filament is labeled with fluorophores and imaged using widefield techniques. Optical tweezers measure the intensity of the force *F*/cos *θ*, while fluorescence allows to estimate both the lengths *h* and *b*; in this way, the *z*-component of the force can be reconstructed. Reprinted with permission from [91].

Equipped with some basic fluorescence vocabulary, we can now embark on an admittedly noncomprehensive overview of recent interesting experiments. One field where combined OT and fluorescence techniques really shine is that of molecular motors. Ishii et al. [91] used a combination of optical trapping, TIRF, and epifluorescence to enrich a standard assay of motility. In Figure 5, a fluorescently labeled thin filament reconstituted from actin, tropomyosin, and troponin is tethered to a fluorescent polystyrene bead and interacts with heavy meromyosin (HMM) molecules attached to the coverslip.

The instrument can switch between TIRF, which images only the region denoted by *a*, and epi-illumination, which allows to visualize also region *b*. The height *h* of the trapped bead with respect to the coverslip is measured from the fluorescence intensity of the bead: the angle *θ* can thus be estimated and the three-dimensional nature of the force vector is properly taken into account. In a somewhat similar OT setup that also makes use of the coverslip as an anchoring point, TIRF has been recently employed to distinguish parallel from antiparallel microtubule bundles [92]. It was therefore possible to ascertain that the mechanical action of the mitotic kinesin Kif15 treats the two cases differently, only sliding apart the antiparallel bundles. Another aspect of mitosis that has been clarified to unprecedented detail is the attachment to microtubules of kinetochores (the molecular machines responsible for chromosome separation). The trap-and-surface geometry OT enhanced by two-color TIRF allowed for clarifying the role played by the central kinetochore component Mis12/MIND in enhancing the microtubule-binding affinity of the external Ndc80 complex [93]. Lin and Ha [94] have published a detailed protocol for building and calibrating an OT integrated with TIRF; they also guide the reader through the steps to setup an experiment about helicase translocation on single-stranded DNA.

**Figure 6:**
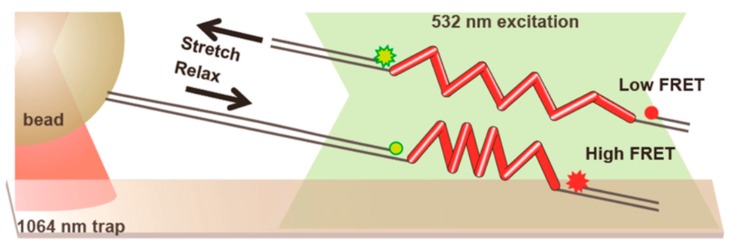
Combination of optical trapping and Förster resonance energy transfer (FRET) in the experimental setup devised by Brenner et al. [95]. The end-to-end extension of the repeat protein made of spider silk peptides is measured by FRET, while optical tweezers are used to apply forces. Trapping laser and fluorescence excitation are kept well apart by long double-stranded DNA (dsDNA) handles. Reprinted with permission from [95]. Copyright 2016 American Chemical Society.

The trap and surface geometry we have described so far lends itself quite naturally to be coupled with widefield fluorescence, especially TIRF due to the role played by the surface, but it has been successfully combined with confocal methods as well. Figure 6, shows a typical setup: FRET intensity varies with the distance between the chromophores, that is the end-to-end extension of the molecule (in this case, repeats of a peptide derived from spider silk), while the optical trap is used to apply and measure forces [95].

Observe that the focal points of the trap and the fluorescence excitation lasers are kept well-separated (around 15 μm for this experiment) to avoid excessive photobleaching: the force exerted by the trap is transmitted by means of long DNA handles. The goal of this research was to study the effect of force on FRET intensity in order to use the peptide repeats as tension sensors in live cell imaging. Regarding instrumental developments, a stimulating new direction has been pioneered by Lee and Hohng [96], who managed to combine optical trapping with three-color FRET. The possibility of simultaneously measuring the mutual distances of three dyes while applying force promises to be a powerful way to address systems of increased complexity.

**Figure 7:**
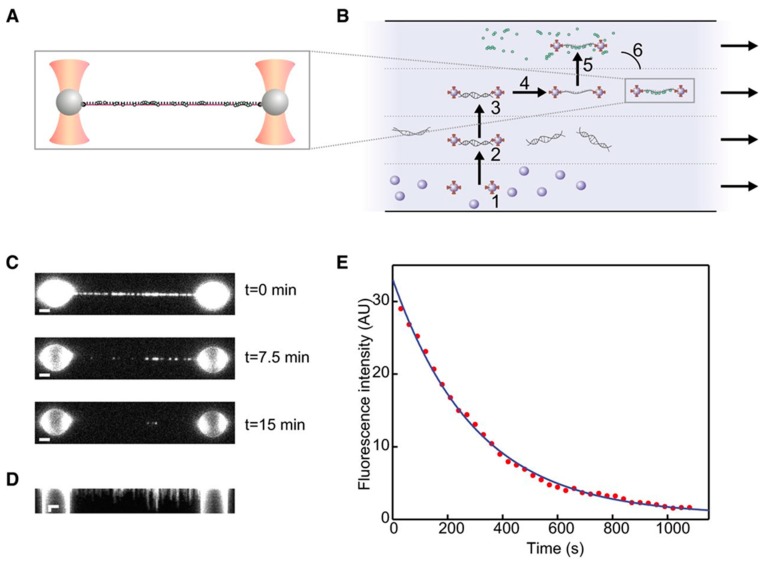
Experiment by Brouwer et al. [97]. (**A**) Dual-trap setup: the molecular construct under study comprises single-stranded DNA (ssDNA) to which fluorescently labeled RAD51 complexes (represented as green dots) are attached. (**B**) Four-channel microfluidic system used to assemble the molecule: the traps capture two polystyrene beads from the bottom channel (1), then a double-stranded DNA (dsDNA) segment is attached to the beads (2), isolated (3), and stretched to form ssDNA (4). RAD51 proteins interact with ssDNA in the top channel (5). Finally, the construct is moved to the free channel where measurements are performed (6). (**C**) From these images obtained by epi-illumination, the fluorescence intensity as a function of time is estimated. (**D**) Kymograph of the same dataset in (C). (**E**) An exponential fitted to the data yields an estimation of the disassembly rate. AU: Arbitrary units. Reprinted with permission from [97].

It is possible, by splitting the trapping laser into perpendicularly polarized beams, to generate two traps that can be independently controlled. Dispensing with a macroscopic anchoring point greatly reduces the thermal drift effects and provides higher resolution OT. The Amsterdam-based group led by Wuite and Peterman is having considerable success in studying DNA repairing molecular machines by coupling the dual trap setup with epifluorescence and a sophisticated microfluidic flow system. A very recent, paradigmatic experiment is illustrated in Figure 7 [97].

The pair of traps is used to move among the four laminar channels to assemble the molecular construct, comprised of single-stranded DNA (ssDNA) and fluorescently labelled RAD51 complexes, which are then imaged using epi-illumination. The integrated fluorescence intensity over time in Figure 7E allows for measuring the disassembly rate, and the experiment can be repeated at different values of tension, as measured by the OT; this provides a persuasive demonstration of the power of combining manipulation and imaging in single molecule experiments. A similar dual-trap plus microfluidics plus widefield fluorescence (both TIRF and epi-illumination) has been used by another group to study RecA, another protein involved in DNA repairing [98]. The integration of high-resolution OT with polarized fluorescence microscopy has been demonstrated in a very recent study [99] of the behavior of YOYO-intercalated double-stranded DNA (dsDNA) subject to forces up to 80 pN, well beyond the DNA overstretching transition. The use of linearly polarized excitation light allowed to check the orientation of the dyes, perpendicular to the axis of the DNA, and to characterize the fast dynamics that they undertake in the time between excitation and emission.

**Figure 8:**
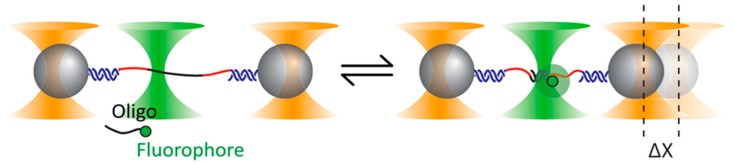
Experimental setup adopted by Whitley et al. [100]. The single-stranded DNA (ssDNA) binding site (black in the figure) is connected by means of two spacers (red) and two double-stranded DNA (dsDNA) handles (blue) to two trapped beads. Binding events of the tagged oligonucleotides are identified by confocal fluorescence. Reprinted with permission from [100]. Copyright 2018 American Physical Society.

If the trapping and the excitation lasers are kept well apart, it is possible to integrate confocal fluorescence into a dual trap OT. A recent demonstration of the advantages of this approach is given by the experiment depicted in Figure 8 [100].

Here the goal is to study the elastic properties of very short (around 10 bp) fragments of dsDNA. To this end, a ssDNA sequence is tethered by long dsDNA handles to two trapped polystyrene beads. Short complementary oligonucleotides are designed to hybridize with segments of the ssDNA, thus forming short fragments of dsDNA. However, the effect of the oligo binding in the low-force regime (around 5 pN) is so small as to lay at the threshold of detectability even for high-resolution OT. The solution: tagging the oligo with a fluorophore and using confocal fluorescence to unambiguously identify binding events. With a similar dual-trap plus confocal fluorescence setup, Ganim and Rief [101] were able to study the response to tension of the green fluorescent protein, establishing that fluorescence is lost right at the outset of an unfolding event and is not recovered until complete refolding. Another confocal fluorescence technique, FRET, excels at revealing small changes in the tertiary structure, and has been profitably coupled to high-resolution dual-trap OT to study the thiamine pyrophosphate riboswitch [102]. The authors managed to observe details about the orientation of a certain helix–arm motif that would have been inaccessible by force or fluorescence spectroscopy alone.

In the examples we have seen so far, the spatial separation between the traps and the confocal fluorescence spot requires the adoption of long dsDNA handles, whose compliance limits the OT resolution. Stiffer, shorter handles grant a better signal to noise ratio, but then the proximity of the trapping light enhances photobleaching. A clever solution is to interlace at high frequency (typically more than 50 kHz) the trapping and the fluorescence excitation lights in such a way that the fluorophore is never simultaneously exposed to both. The sophistication of the instrumental design is more than compensated by the increase in resolution; see, for instance, the very detailed reconstruction of the energy landscape of ssDNA binding protein obtained by the authors of [103]. Whitley et al. [104] have recently published a thorough description of the instrumental design of an interlaced OT/FRET setup, with a guide to alignment procedures and tutorial instructions to perform an experiment on helicase UvrD. An alternative design proposed by Sirinakis et al. [105] offers great versatility, combining in a single instrument dual trap OT with either widefield or confocal fluorescence.

The success of the approach based on joint force spectroscopy and fluorescence imaging is not limited to in vitro experiments. In their study of the mechanical properties of filopodia, Leijnse et al. [106] used confocal scanning microscopy to image F-actin in living HEK293 cells, and optically trapped beads to manipulate single filopodia. This allowed them to reveal rotational motion and helical buckling of the actin shaft inside retracting filopodia, a mechanism that had previously gone unnoticed. A similar approach (scanning confocal three-dimensional imaging accompanied by OT-induced manipulation) was applied to the study of adhesion between cells and biomaterial scaffold [107]. The remarkable observation that the cell growth during the first week of culture is strongly correlated to the adhesion forces measured within a few seconds after the first contact suggests a quick way of predicting scaffold biocompatibility, with possible impacts on the field of regenerative medicine. In conclusion, we would like to mention a recent experiment in which Pang et al. [108] managed to optically trap and image (using two-photon fluorescence) single human immunodeficiency virus-1 (HIV-1) virions in culture fluid, which presents a powerful new technique for virology.

## 6. Conclusions

The advent of optical trapping techniques in the sphere of medical science and biophysics has been accompanied by the exploration of new horizons in the optical tweezers technology. In this paper we illustrate and discuss some of the latest versions of OT, highlighting their instrumental features and biological applications. While these techniques are opening new avenues of optical manipulation, a salient feature common to all of them is the vast potential for further improvement, especially when dealing with biomaterials. POT have facilitated efficient optical trapping in the Rayleigh regime, making small biomolecules a fair game for optical trapping experiments [51,52]. Yet, controlled probing and manipulation capabilities by means of plasmonic traps are still limited. Moreover, photothermal effects observed during plasmonic trapping diminish the efficiency of this technique as they create adverse local thermophoretic gradients [45]. Integrating POT with lab-on-chip devices, like surface plasmon resonance (SPR)-based biosensors [109] and surface acoustic waves (SAW) resonators [110], might allow us to explore different schemes of manipulation and enhance the investigation power of this technique. Similarly, theoretical studies in the field of thermal convection [111] and employment of novel material and nanostructure geometries [45] might help us develop new strategies to curb the shortcomings due to thermal effects. Femtosecond OT and PhC OT can be extremely effective for in vivo manipulation experiments as they cause little photothermal damage to the cells [61,73]. Combining this optical trapping capability with the tools at the disposal of lab-on-chip devices will most likely offer a powerful method to investigate biophysical properties of living cells [61,73]. On the other hand, hybrid systems combining optical traps with fluorescence techniques are so advanced from an instrumental viewpoint that at the moment of writing there are two readily available commercial solutions (JPK Nano Tracker™ and LUMICKS C-Trap™) that integrate optical manipulation with several kinds of fluorescent imaging. As technical problems are being resolved, research groups are shifting their attention from the instrument to its ingenuous biological applications, making optical trapping an increasingly powerful tool in life science studies, a fitting homage to the seminal paper [1] that, more than 30 years ago, started the single-molecule revolution.
